# Brief reviews

**DOI:** 10.1590/S1980-57642012DN06010012

**Published:** 2012

**Authors:** Sonia Maria Dozzi Brucki

## HIPPOCAMPAL SCLEROSIS IN THE ELDERLY: GENETIC AND PATHOLOGICAL FINDINGS, SOME MIMICKING ALZHEIMER'S DISEASE CLINICALLY

**Pao et al.** Alzheimer Dis Assoc Disord 2011;25:364-368.

Hippocampal sclerosis (HS) is a pathological diagnosis characterized by selective neuronal loss and gliosis without cystic cavitation in the subiculum and CA1 sector of the hippocampus. HS can be diagnosed in AD when neuronal loss is disproportionate to the density of neurofibrillary tangles (NFT), in particular, the presence of extracellular NFTs.

These authors reported 205 patients with dementia who had been prospectively evaluated at the Mayo Clinic; 43% were women and the median age at death was 79 y (range: 37 to 99 y).

Twenty-eight patients (14%) had HS, of which 25 (89%) had TDP-43 positive inclusions, compared to positivity in 24% of patients without HS.

HS was present in 65% of cases with FTLD-U; in 22% of AD cases with TDP-43 pathology, and in 2% of AD cases without TDP-43 pathology.

Among patients who had HS with TDP-43 pathology and onset of dementia before 75 y: six presented with features of FTD and seven had predominant amnestic features (clinically diagnosed as AD). For cases with HS and TDP-43 pathology with dementia onset after 75 y (n=11), eight had an amnestic syndrome (clinically diagnosed as probable AD), and six had Braak stage III or less.

In summary, HS was present in older patients, in those whose dementia began after 75 y of age, and the most common presentation was probable AD. The GRN (progranulin) rs 5848 T-allele but not APOE E4 was associated with HS.

## ACCELERATED CORTICAL ATROPHY IN COGNITIVELY NORMAL ELDERLY WITH HIGH b-AMYLOID DEPOSITION

**Chételat et al.** Neurology 2012;78:477-484.

Aβ deposition on PIB-PET can be seen in approximately one third of cognitively normal elderly individuals. It is still not clear whether an Ab-positive pattern in asymptomatic individuals should be considered pathologic.

Authors compared the rate of regional brain atrophy over 18 months between normal elderly persons with PIB positive (PIB+) and PIB negative (PIB-) scans. MRI (3T-scanner) and PIB-PET were obtained at inclusion, and a second MRI was performed 18 months later.

Seventy-four normal healthy elderly persons (54 PIB- and 20 PIB+) comprised 50% subjects with subjective memory complaints and 50% ApoE4 carriers. There was no difference in cognitive performance by PIB groups; overall, PIB+ subjects were older than PIB-. During the follow-up period, one participant converted to AD and three to MCI (all PIB+).

The average rate of atrophy was 0.005 mm^3^/voxel/y in the PIB- group and 0.0065 mm^3^/voxel/y in the PIB+ group, corresponding to 0.5% and 0.65% loss per year, respectively. There was a significant positive correlation between increased rate of atrophy and increased neocortical PIB deposition. Analysis revealed a significant difference in the annual percent volume loss between PIB- and PIB+ in the middle, superior and inferior temporal cortex and posterior cingulate-precuneus.

## REVISED CRITERIA FOR MILD COGNITIVE IMPAIRMENT MAY COMPROMISE THE DIAGNOSIS OF ALZHEIMER DISEASE DEMENTIA

### **Morris JC.** Arch Neurol doi: 10.1001/archneurol.2011.3152

According to Dr. Morris the categorical distinction between MCI and milder stages of AD dementia has been compromised by the revised criteria of the National Institute of Aging and Alzheimer's Association.

Participants were enrolled at Federally funded Alzheimer's disease centers (ADC) and were assessed by two ratings of activities of daily living, namely, the Functional Activities Questionnaire (FAQ) and the Clinical Dementia Rating (CDR) scale; subjects were classified as MCI, very mild or mild dementia (all diagnoses were reached prior to the publication of the reviewed criteria for MCI).

Participants were diagnosed as being cognitively normal (n=6379), having MCI (n=4947), or probable AD (n=6209) between September 2005 and May 2011. Subjects had a mean age of 74.6 (9.5) y, mean educational attainment of 14.7 (3.5) y; 59% were women and 41.7% were carriers of at least one E4 allele.

Observing the two ratings of functionality, 99.8% individuals currently diagnosed with very mild AD dementia and 92.7% of those diagnosed with mild AD dementia, were reclassifiable as having MCI based on the revised criteria.

The revised criteria for MCI rendered the distinction between MCI and AD unclear by allowing mild difficulties in functional activities. The vast majority of individuals diagnosed with milder stages of AD dementia became reclassifiable as having MCI under the revised criteria. The author highlighted the influence of personal opinion in electing functional activities allowed.

